# Unveiling the Role of Dps in the Organization of Mycobacterial Nucleoid

**DOI:** 10.1371/journal.pone.0016019

**Published:** 2011-01-24

**Authors:** Payel Ghatak, Kajari Karmakar, Sanjay Kasetty, Dipankar Chatterji

**Affiliations:** 1 Molecular Biophysics Unit, Indian Institute of Science, Bangalore, India; 2 Friedrich Miescher Institute for Biomedical Research, Basel, Switzerland; University of Hyderabad, India

## Abstract

In order to preserve genetic information in stress conditions, bacterial DNA is organized into higher order nucleoid structure. In this paper, with the help of Atomic Force Microscopy, we show the different structural changes in mycobacterial nucleoid at different points of growth in the presence of different concentrations of glucose in the medium. We also observe that in *Mycobacterium smegmatis*, two different Dps proteins (Dps1 and Dps2) promote two types of nucleoid organizations. At the late stationary phase, under low glucose availability, Dps1 binds to DNA to form a very stable toroid structure. On the other hand, under the same condition, Dps2-DNA complex forms an incompletely condensed toroid and finally forms a further stable coral reef structure in the presence of RNA. This coral reef structure is stable in high concentration of bivalent ion like Mg^2+^.

## Introduction

The *Mycobacterium smegmatis* mc^2^155 strain has a single molecule of covalently closed circular DNA comprising of 6,638,000 base pairs with linearized length of 2.32 mm (http://www.tigr.org/tdb/mdb/mdbinprogress.html), or diameter of 0.74 mm when it exists as a relaxed circle. During stationary phase of growth cycle, the approximate diameter of mc^2^155 cell is 2.5–3.5 µM, immediately indicating a great deal of compaction of the genome (∼1000-fold), in order to accommodate it within a single cell [1]. The higher order structure of DNA and its compaction assisted by different proteins in eukaryotes is a well-researched area with enormous literature [2], [3]. In prokaryotes, the field is developing and the role of different DNA binding proteins in organized DNA structures are being increasingly understood with interesting manifestations [4]. The nucleosome structure is the hallmark of eukaryotic genome organization [3], [5]. However, in prokaryotes such organized nucleosomes are non-existent; rather, formation of nucleoid governs the genome architecture and packing. In *Escherichia coli*, the nucleoid formation is greatly facilitated by 12 histone like DNA binding proteins HU, IHF, HNS etc. [4], [6]–[9]. The fundamental structural organization of nucleoid has been worked out recently with the help of Atomic Force Microscopy (AFM) [10]. It has been shown that a protein Dps (DNA binding protein under starvation) plays a major role in tight compaction of nucleoid in *E. coli* [11].

Although some of the DNA binding proteins in *M. smegmatis* have been reported recently, a complete account is still not available. There are close to 7000 genes in *M. smegmatis* and one of the major DNA binding proteins is Dps [12]. The role of Dps is currently being worked out at different laboratories and we have observed interesting role of Dps in protection of DNA from reactive oxygen species [11], [13]–[16]. However, the function of these DNA binding proteins in nucleoid organization in *M. smegmatis* has not been addressed properly. In a recent report, it has been proposed that in a growing *E. coli* culture, the genome undergoes a massive reorganization in stationary phase with ordered toroid structure [10], [17]. This transition is a necessity at the stationary state in order to make the organization less dependent on energy progressively.

We have reported before, that *M. smegmatis* has two Dps molecules, Dps1 and Dps2, [15], [18]. The former is required to protect DNA in bimodal fashion and express predominantly at the stationary phase, whereas the later express constitutively and appears to play a role in DNA packing [19], [20]. In this paper, along with the time dependent changes in nucleoid structure, we have tried to show that Dps1 and Dps2 form two types of nucleoids in *M. smegmatis*. We have mainly used Atomic Force Microscopy in this study. Interestingly, both organized structures have been formed at stationary phase or under depletion of carbon and they look like toroids. However, the toroid formed by Dps2 further converts into more stable coral reef structure with the participation of RNA. For elucidating the role of Dps2 in coral reef formation, we have constructed the Dps2 deleted strain of mc^2^155 and observed the absence of such stable coral reef structure in it.

## Results

### A) Atomic force microscopic studies on *M. smegmatis* mc^2^155

**Figure 1:**
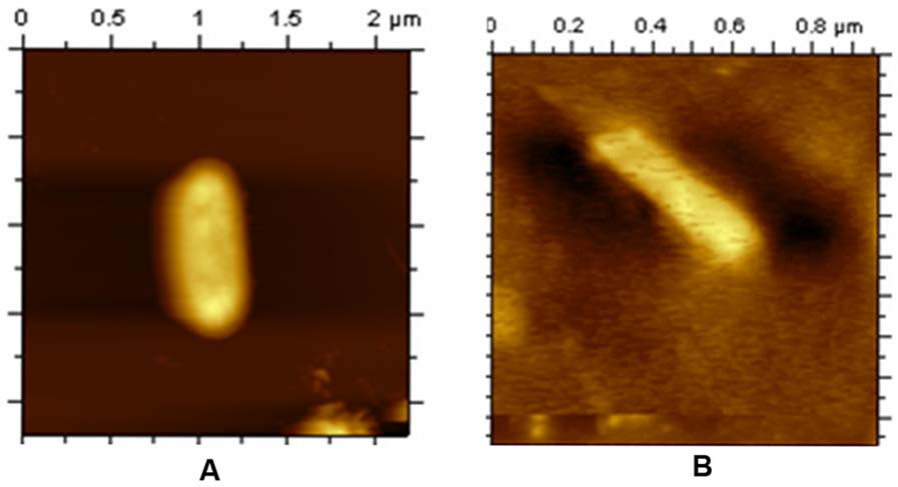
AFM images of cells of *Mycobacterium smegmatis*. (A) Structure of bacterial cell before on-substrate lysis treatment. (B) Structure of bacterial cell after on-substrate lysis treatment.

We have noticed that all previous AFM analysis of bacteria was carried out upon lysing the cells on the surface of mica prior to image analysis [21]–[23]. However, any such attempt with mycobacteria was not successful due to the presence of very tough cell wall [24]. At first, we had followed the “On-substrate lysis” procedure as described earlier [22]. However, we were unable to lyse the mycobacterial cells (Figure 1). Hence, we had to lyse the cells by sonication before spreading them over freshly pealed mica for imaging and the complete procedure is described in Materials and methods section. Over expressed Dps2 cells were harvested up to 170 h and 220 h. Two different types of images were obtained by following the above mentioned lysis procedure. One of them (cells grown up to 170 h) showed nucleoid, compacting from a nonspecific structure to a circular one (Figure S1A). The other (grown up to 220 h) showed spherical shape and its diameter is comparable with the previous reports regarding bacterial nucleoid size (Figure S1B) [25]. It has also been reported that at late stationary phase of growth the nucleoid in *E. coli* becomes stable to break with “on substrate lysis” procedure [10]. Our above observation supports this report. In all cases of lysis and subsequent AFM studies, equal number of cells of O.D. 0.5 was taken.

### B) DNA organization by Dps1 and Dps2

Following experiments described here will address several issues, like;

a) Nature of the nucleoid from *M. smegmatis* under different stages of growth (12 h, 24 h, 48 h, 72 h, and 144 h) i.e. from early log phase to late stationary phase. b) Characteristic of DNA structure when either Dps1 or Dps2 are over expressed, and c) nature of the DNA condensation when either of the Dps protein is deleted from the cells. In this case, we were unable to generate a strain of *M. smegmatis* with deleted Dps1 and therefore our experiments were limited only to Dps2 deleted *M. smegmatis*. In addition, we also looked at the role of RNA in nucleoid organization by treating the nucleoid with an ssRNA degrading enzyme RNaseA. All AFM images have been repeated 30–40 times in different scanning zones and showed reproducibility within error limit.

#### B (i)

Four types of mycobacterial strains [wild type, over expressed MsDps2 (*M. smegmatis* Dps2 protein), over expressed MsDps1 (*M. smegmatis* Dps1 protein), Knockout MsDps2 (KK09)] were grown in different time (12 h, 24 h, 48 h, 72 h and 144 h) in both 2% and 0.02% glucose (Figure S2). Throughout here, we have described 12 h, 24 h, 48 h, 72 h, and 144 h of growth in 2% glucose as early log phase, log phase, early stationary phase, stationary phase, and late stationary phase, respectively. However, in 0.02% glucose containing medium, mycobacterium reaches stationary phase at 48 h and very late stationary phase at 144 h.

**Figure 2:**
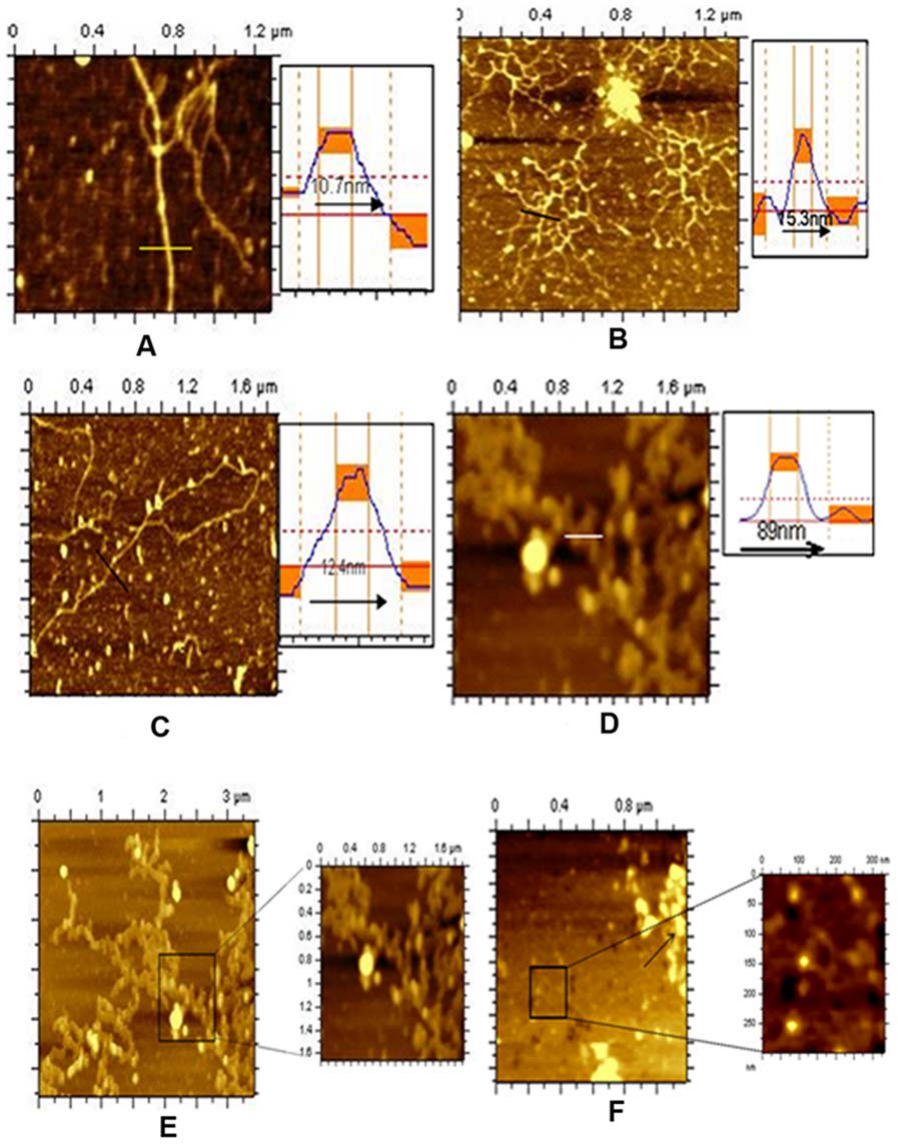
Nucleoid structures of wild type mc^2^155 strain grown in MB7H9 media containing 2% and 0.02% glucose. (A) Nucleoid structure of wild type mc^2^155 cells, which were grown in 2% glucose up to 12 h (intramolecular contact numbers are 2-4). (B) Nucleoid structure of wild type mc^2^155 cells grown up to 144 h in 2% glucose (intramolecular contact numbers are 60-70). (C) Nucleoid structure of wild type mc^2^155 cells grown in 0.02% glucose up to 12 h. (D) Nucleoid structure of wild type mc^2^155 cells grown until 144 h in 0.02% glucose. The solid lines in AFM images represent the sites of section analysis that were depicted in the right upper panels. The horizontal dotted line on section analysis indicates the half-maximum height. The peak-to-peak distance of DNA in AFM images (A, B, C and D) were measured and appeared to be 10.7 nm (10±2.5 nm) (A), 15.3 nm (15±1.4 nm) (B), 12.4 nm (11±2.5 nm) (C) and 89 nm (85±7.8 nm, mean±SD) (D). (E) It represents the extended version of Figure 2D. (F) Toroid and a part of coral reef structure from wild type *M. smegmatis* cell, grown up to 144 h in 0.02% glucose. Box shows the toroid and arrow shows a part of coral reef structure.

Figure 2 (A–D) shows the AFM images of the lysate from the wild type mc^2^155 cells at 12 h and 144 h of growth, when grown in 2% (A, B) and 0.02% (C, D) glucose containing MB7H9 media. It can be seen from the Fig. 2A that DNA remained free at 12 h (intramolecular contact no. 2–4) whereas, at stationary phase (72 h) it was in DNA bound form i.e. each nucleoid has an increased number of intramolecular contacts (intramolecular contact no. 60–70) [Figure 2B] [26]. On the other hand, in glucose depleted media, DNA was free at early log phase (12 h) [Figure 2C] but formed DNA-protein organized complex from stationary phase (48 h) and will continue to late stationary phase also (72 h) [Figure 2D]. Here the number of intramolecular contacts becomes very high at late stationary phase (72 h) and after certain time (144 h), we cannot distinguish DNA in DNA-protein complex. This structure is known as coral reef structure (Figure 2E). One can immediately notice a change in DNA organization [Figure 2B and 2D] as a function of glucose concentration, at the same time of growth in different medium (72 h). Figure 2E represents the same sample as Figure 2D, at finer resolution.

**Figure 3:**
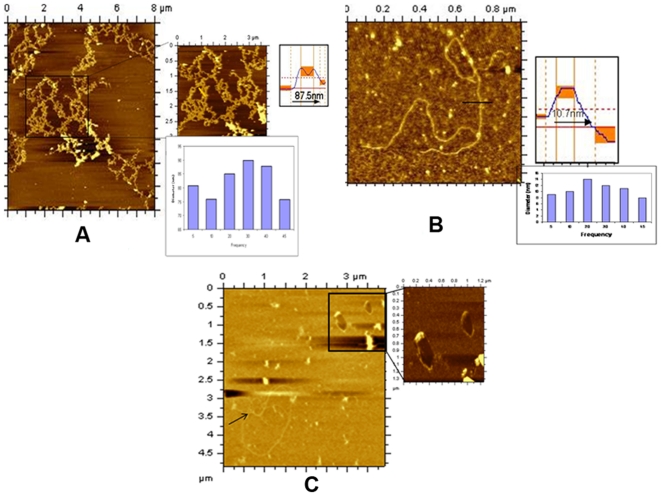
Overall nucleoid structure formed by over expressed Dps2, knockout MsDps2 (KK09) and over expressed Dps1 strain. (A) Coral reef structure formed by over expressed MsDps2 strain at 144 h in 0.02% glucose. The *msdps2* gene was cloned into a mycobacterium over expression vector to generate pAGAN *msdps2*, and then *M. smegmatis* cells were transformed with pAGAN *msdps2* (See Materials and methods). The diameter of the fiber in coral reef is 87.5 nm (83±8 nm, mean±SD). The histogram shows the statistical value of experimental data. (B) Overall nucleoid structure of Dps2 Knockout strain of mc^2^155 at 144 h in 0.02% glucose**.** A recombination cassette of 3.4 kb was constructed to delete MSMEG_3242 from *M. smegmatis* mc^2^155 (Text S2). The solid lines in images represent the sites of section analysis that are depicted in the upper right corner of the image. The horizontal dotted line on section analysis indicates the half-maximum height. The peak-to-peak distance of nucleoid in this image was measured and appeared to be 10.7 nm (12±2 nm mean±SD). The histogram supports the statistical values. (C) Nucleoid from *M. smegmatis*, when Dps1 was over expressed. Cells were grown up to 144 h in MB7H9 media containing 0.02% glucose and then were lysed and further analyzed by AFM. The diameter of each toroid is 40 and 45.3 nm (42±5 nm, mean±SD). Arrow shows the DNA-protein complex.

We have shown before, that glucose starvation in *M. smegmatis* elicits several responses, which are close to that of latent or persistent cells both by appearance as well as at the genetic level [27], [28]. Next, we wanted to find out the role of Dps2 in the organization of DNA in *M. smegmatis*. When Dps2 was over expressed at 2% glucose, the nature of the DNA-protein complex was different from the one grown in 0.02% glucose. The former one appears to be linear DNA complexes with protein; whereas, a very clear coral reef organized nucleoid was visible at low glucose condition (Figure 3A).

#### B (ii)

Coral reef pattern formation is unique for bacterial nucleoid as reported earlier and we observed such patterns when cells were either grown up to very late stationary period (144 h) under glucose depleted condition (0.02%) (Figure 2E) or when MsDps2 was over expressed (Figure 3A). We estimated that the diameter of each thread of the DNA-protein complex in coral reef structure was 80–90 nm. In addition, when immunoprecipitation was carried out with Dps2 antibody (Text S1), lysate made from Dps2 over expressing cells showed the presence of Dps2 indicating the role of this protein in nucleoid organization (Figure S3).

In order to establish this connection, we generated a strain of *M. smegmatis*, which is devoid of Dps2 gene and named this knockout strain as KK09. When this strain was grown until 144 h in 0.02% glucose, no coral reef was observed expectedly, and one can notice the DNA-protein complex (Figure 3B). In this case, the diameter of each thread of DNA-protein complex is 10–15 nm. Interestingly, when Dps1 was over expressed and grown for 144 h in low glucose, we did not observe any coral reef structures in the lysed cells, rather we found toroid like structures [Figure 3C].

### C) Role of RNA in genome organization

It has been reported earlier that transcription machinery of the cell or RNA plays an important role in bacterial nucleoid as well as during coral reef formation [10], [21], [25], [29]–[32]. The role of RNA can be detected by treatment with RNaseA, which will destroy the coral reef structure. On the other hand, addition of rifampicin to growing cells (at 72 h) inhibits the synthesis of RNA, which would interfere with the formation of desired nucleoid structure (Figure S4).

**Figure 4:**
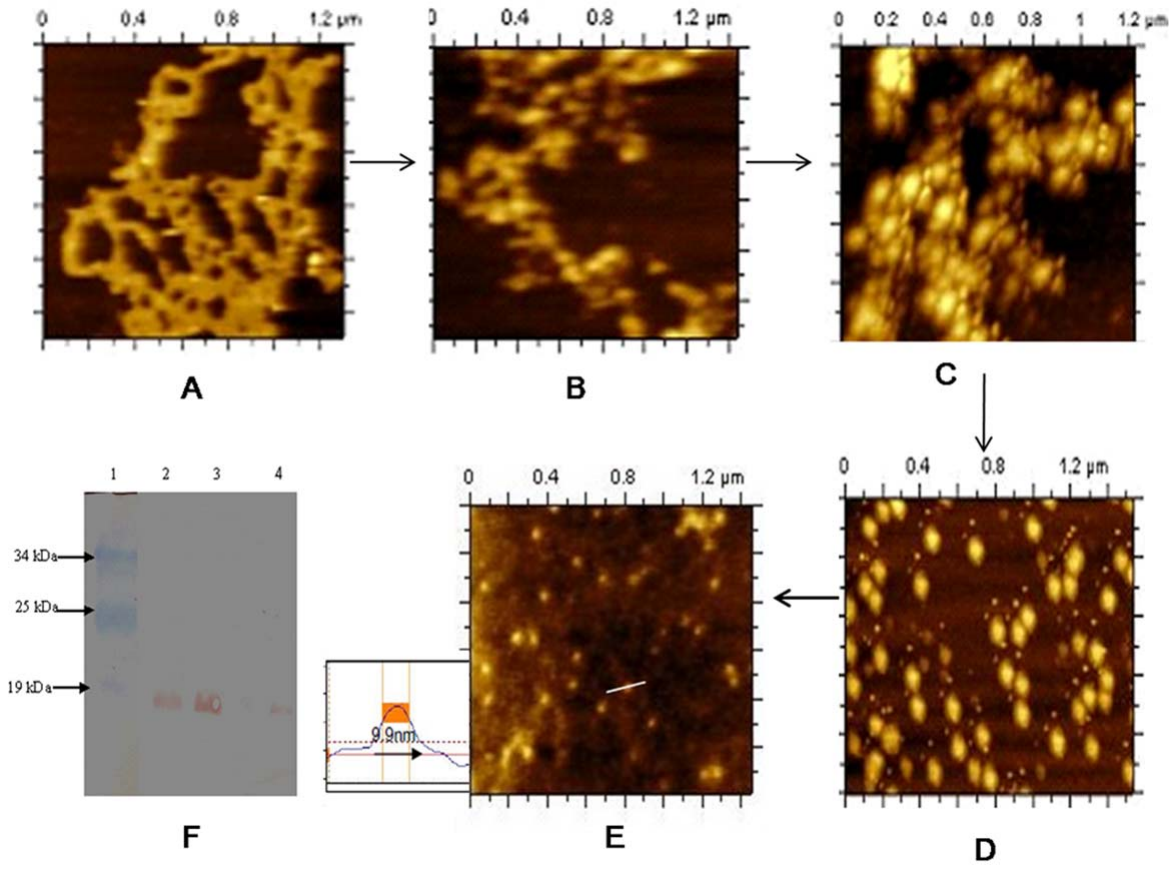
Gradual destruction of coral reef nucleoid structure in *M. smegmatis* with increasing concentration of RNaseA and western blot analysis. (A–E) The mc^2^155 cells over expressing Dps2 were grown up to 144 h in 0.02% glucose. The same sample was treated with different concentration of RNaseA from 0.5 mg/ml, 1 mg/ml, 2 mg/ml, 5 mg/ml, (B to E) respectively. One may notice that in Figure 4E only Dps2 protein was detected [diameter 9.9 nm (8.5±5 nm, mean±SD)]. The solid lines in images represent the sites of section analysis that are depicted in the upper right corner of the image. The horizontal dotted line on section analysis indicates the half-maximum height. (F) Western blot analysis of over expressed Dps2 protein (with or without RNaseA treatment). Lane 1; shows prestained protein Marker. Lane 2; lysate without RNaseA treatment (control) and Lane 3; shows Dps2 with 5 mg/ml RNaseA. Lane 4 shows pure Dps2 protein. The molecular weight of each is 19 kDa.

With increasing concentration of RNaseA, the coral reef structure of DNA-Dps2 progressively disappeared as shown in (Figure 4A–E). Figure 4E presumably represents only the protein molecule with diameter ∼9 nm. When the same sample was loaded onto 15% SDS-polyacrylamide gel, electrophoresed and transferred to nitrocellulose membrane, the protein was identified as Dps2 (Figure 4F). The above experiment established that RNA is present in the coral reef structure and its removal destabilizes the nucleoid assembly. It should be mentioned here that we did not see any structural changes in MsDps1-DNA complex after the addition of RNaseA (Figure S5).

### D) Effect of Magnesium ion on DNA-protein complex

It has been reported earlier that high concentration of Mg^2+^ ion (>7.5 mM) inhibits Dps- DNA complex formation in *E. coli* [33], [34]. In our experiment, we have used three different concentrations of Mg^2+^ ion like, 1 mM, 7.5 mM, and 10 mM. In over expressed Dps1 strain, the nucleoid structures were more or less same at the concentration of 1 mM and 7.5 mM Mg^2+^ ion [Figure S6A (1–2)]. However, a drastic conformational change was found with the addition of 10 mM Mg^2+^, when the structures converted from a highly complex structure to that of a free DNA [Figure S6A (3)]. Similar results were obtained in the case of nucleoid from mutant Dps2 strain (KK09) [Figure S6B]. On the contrary, in over expressed Dps2 strain, there were no significant changes in coral reef structure at different Mg^2+^ concentration [Figure S6C]. It should be mentioned here that the Dps1- toroid structure did not change with the addition of 10 mM Mg^2+^, as shown in [Figure 3C].

### E) Toroids by Dps1 and Dps2

**Figure 5:**
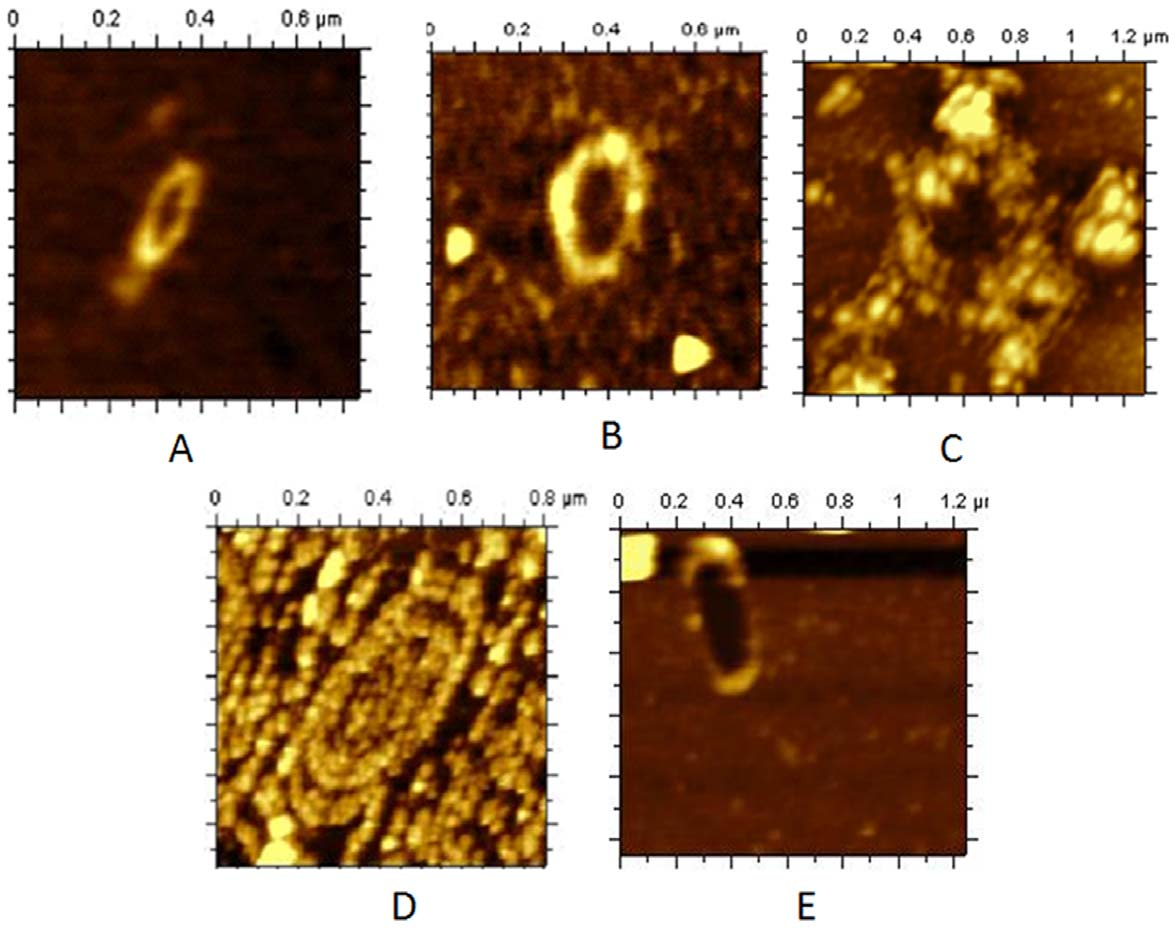
Effect of RNaseA on toroid structures formed by Dps1 and Dps2. (A–B) Toroid structure formed by Dps1 before and after RNaseA treatment. mc^2^155 cells with over expressed Dps1 were grown until 144 h in MB7H9 media containing 0.02% glucose. The lysate shows toroid before (A) and after (B) 5 mg/ml RNaseA treatment. In Figure A, the diameter of the toroid varies between 50–60 nm, whereas in Figure B, it varies between 70–100 nm. In several such estimation of different toroids the mean value ranges as 80±30 nm, mean±SD. (C) Toroid structure formed by Dps2 after RNAseA treatment in over expressed Dps2 strain grown up to 144 h at 0.02% glucose. Diameter of the outer sphere is 200±50 nm, mean± SD. (D–E) Toroid structures formed by purified Dps2 [Figure D] and purified Dps1-pGEM-7Zf (+) [Figure E]. Both proteins were purified as discussed in Materials and methods. The pure proteins were subjected to AFM analysis and toroid has a diameter varying between 50–100 nm for Dps1 and 200–250 nm for Dps2.

According to earlier reports, DNA bound Dps in *E.coli* forms toroid structure. In wild type *M. smegmatis* cell, we have found toroid structure as well as coral reef structure (Figure 2F) when they grow in 0.02% glucose at very late stationary phase (144 h). Cells with over expressed MsDps1 shows clear toroids (Figure 5A), which are not susceptible to RNaseA (Figure 5B). We have seen toroid structure in over expressed MsDps2 cell lysate grown in 0.02% glucose (Figure 5C). However, the structure of the toroid, formed by Dps2, is distinct from that of Dps1.

To confirm our report that these toroids were formed only by Dps1 and Dps2 and not by other proteins in the cell, we carried out in vitro experiments. Here we used pure proteins and added plasmid DNA. We reported earlier, that MsDps2 is purified from cells in DNA bound form [20]. Hence we used only purified Dps2 protein, and, on the other hand, we added plasmid pGEM-7Zf(+) to pure Dps1 protein to generate toroids (Figure 5D and 5E respectively). Upon comparing, we observed that Dps2 forms toroid with 200–250 nm diameter which is larger than that of Dps1 (50–100 nm).

**Figure 6:**
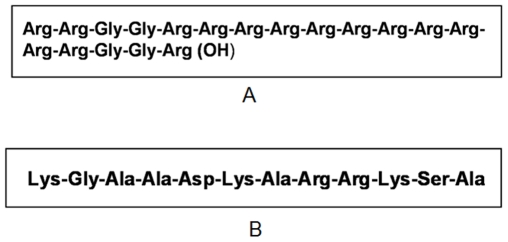
C-terminal sequences of protamine and MsDps1. (A) shows the C-terminal sequence of protamine. (B) shows C-terminal sequence of Dps1. Both have a large number of basic amino acids (lysine, arginine).

In our study, we found that the physiological structure of Dps1- DNA toroid is very similar to that of protamine- DNA toroid [35], [36]. Hence, we were interested to compare these two classes of toroids. We proposed earlier that MsDps1 binds DNA through its C-terminal end [37]. Upon closer examination, we found that protamine has a stretch of basic amino acids in its C-terminal end similar to the C-terminal tail of MsDps1 (Figure 6). Thus, we can propose here that a tandem repeat of positively charged amino acids is necessary to generate this type of toroid structure. Such type of toroids of bacterial Dps protein has not been demonstrated before. On the other hand, Dps2 does not have any repeat of positively charged amino acids in either end and it appears that DNA wraps around the protein with the help of RNA generating different forms of toroid at the stationary phase, which converts to coral reef upon prolonged incubation [18], [19].

## Discussion

It was reported before that both toroid and coral reef are the “last resort for survival” of a bacterial species under stress [11], [38]. However, there are two types of toroids that can be seen in bacteria. Toroid with diameter less than 100 nm is extremely stable [9], [40]. On the other hand, toroids with larger diameter (>200 nm) are unstable [40]–[42].

In our study presented here, we have observed that two types of toroids can be seen in mycobacterial nucleoid. Smaller toroid, the stable one, is found in over expressed Dps1 cells and does not form coral reef. The other type or the larger toroids are generated during over expression of Dps2. For further stability, this type of toroid converts into coral reef structure in the presence of RNA. Both the smaller toroid and coral reef structure are very stable and protect the genomic DNA in stress condition. However, it is very difficult for us to predict at this stage, which is more transcriptionally active.

**Figure 7:**
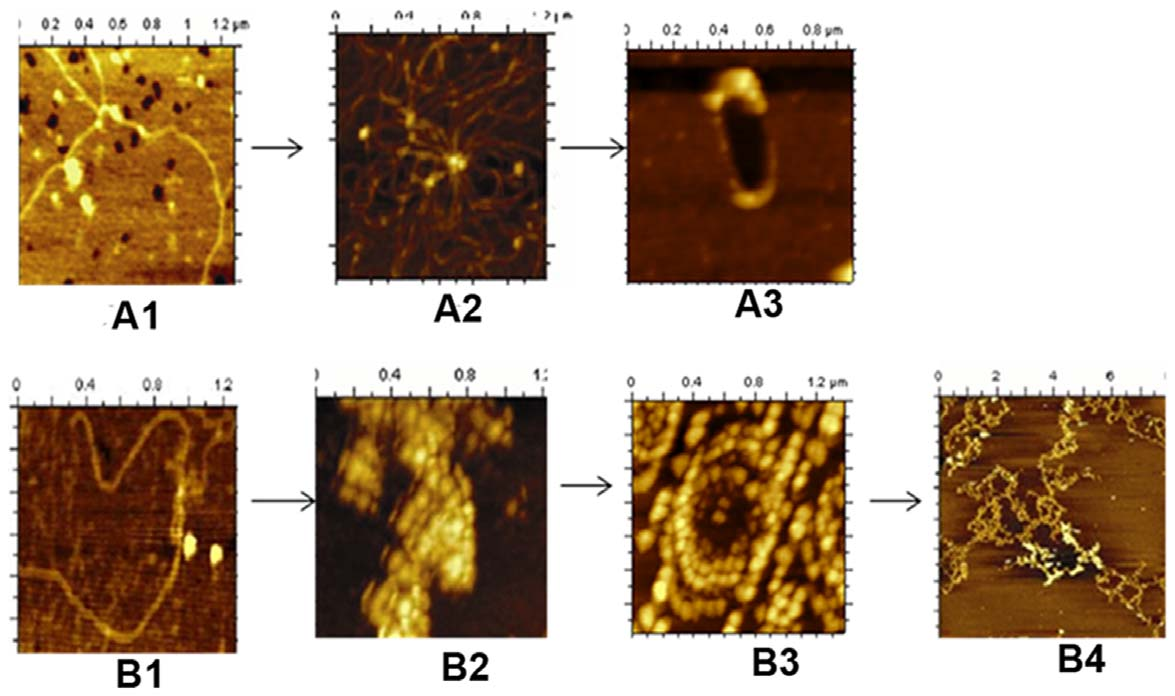
The overall nucleoid organization in *M. smegmatis.* A (1-3) Nucleoid organization by Dps1 protein from free DNA to toroid. (1) Free DNA, (2) Dps1-DNA in log phase (24 h), (3) Dps1- toroid in late stationary phase (144 h). B (1-4) Nucleoid organization by Dps2 protein from free DNA to Coral reef. (1) Free DNA, (2) Dps2-DNA complex in log phase (24 h), (3) Dps2- toroid in late stationary phase (72 h), (4) Coral reef structure in very late stationary phase (144 h).

DNA organization in Gram-positive bacteria has not been studied very well so far. In this paper, we propose two different paths for DNA organization in *M. smegmatis* by two different Dps proteins. Figure 7A and 7B compare these organization schematically where one set of organization is guided by the tandemly distributed positively charged amino acids present in the C-terminal of Dps1 leading to the formation of toroid. On the other hand, Dps2, assisted by RNA, first generate a different class of toroid, which finally form coral reef structure in late stationary phase. We have also shown that this coral reef structure is so stable that it can retain itself even in the presence of high concentration of bivalent ions like Mg^2+^.

It has been reported recently that the nucleoid-associated proteins have a role in gene expression [7]. We know that the transcription of MsDps2 is regulated by σ^B^, which is analogous to σ^s^ in *E.coli* [20]. The *rpos* gene, that is sensitive to post-transcriptional modification, regulates the sigma factor σ^s^ [7], [43]. We would like to speculate that MsDps2 as a nucleoid-associated protein may also have an effect on post-transcriptional modification [20].

Although we have presented here DNA organization in non-pathogenic mycobacteria, the results may have some relevance with other pathogenic species of mycobacteria. For an example, it has been shown that a GroEL homolog in *M. tuberculosis* is nucleoid-associated and may substitute other DNA binding proteins like Dps which are absent in this organism [44]. Upon comparing both Dps2 (*M. smegmatis*) and GroEL1 (*M. tuberculosis*) we have observed some remarkable similarities. Both of them can bind DNA without sequence specificity, both are nucleoid-associated proteins with comparable binding affinities to DNA. In both cases, protein-DNA complexes showed different degree of condensation. In addition, they can bind RNA too. Thus, it will be interesting to follow the state of nucleoid in *M. tuberculosis* in the absence of this homolog of GroEL.

## Materials and Methods

### Bacterial strains and growth conditions

All the plasmids and strains used in this study have been listed in Table 1.

**Table 1:** Strains and plasmids used in the study.

Strains and plasmids	Description	Reference or Source
*M. smegmatis* mc^2^155	Parental strain	This study
MsDps2 KO (KK09)	Dps2 Knockout mc^2^155 strain	This study
pAGAN *msdps1*	MsDps1 over expressed in pAGAN	Saraswathi R (Ph.D. Thesis)
pAGAN *msdps2*	MsDps2 over expressed in pAGAN	Saraswathi R (Ph.D. Thesis)
pGEM7Zf(+)	2.9 kb *E. coli* cloning vector, *lac*Z, *amp* ^r^, f1 origin.	Promega corp.
pUC4K	3.9 kb *E. coli* vector, source of *kan* ^r^ gene.	Pharmacia Biotech
pPR27	*E. coli* – Mycobacterial shuttle vector, *ts oriM*, *sacB*, *gm* ^r^.	[47]
pMD2UP	1 kb upstream region of msdps2 cloned into *Xba*I*-Eco*RI sites of pGEM-7Zf (+) vector.	This study
pD2DN	1.2 kb downstream region of *msdps2* cloned into *Eco*RI*-Bam*HI sites of pMD2UP.	This study
pPRD2KO	3.4 kb construct containing upstream and downstream region of *msdps2* with *kan* ^r^ gene, cloned into pPR27 between *Xba*I sites.	This study
pMV261	Cloning vector with hsp60 promoter, *kan* ^r^	[48]

The BL21 (DE3) plysS strain of *E. coli* Genotype: F^−^, ompT, gal, dcm, lon, hsdsβ (r_β_^−^ m_β_^−^, an *E. coli* B strain with DE3, a λ prophage carrying the T7 RNA polymerase gene) was used for protein purification. For wild type cell lyses, *M. smegmatis* wild type strain mc^2^155 was grown in MB7H9 medium supplemented with 2%, 0.02% glucose and 0.05% Tween-80. In Over expressed MsDps1, the *msdps1* gene was cloned into a mycobacterial over expression vector to generate pAGAN *msdps1*, and then *M. smegmatis* cells were transformed with pAGAN *msdps1*. We have followed same procedure for over expressed MsDps2 strain; here the vector is pAGAN *msdps2*. In case of *msdps2* knockout, we constructed a recombination cassette to delete *MSMEG_3242* from the chromosome of *M. smegmatis* mc^2^155. The cassette consisted of a 3.4 kb construct containing upstream and downstream regions of *msdps2* with an *Eco*RI insert of pUC4K containing the *kan* ^r^ gene cloned into *Eco*RI site of pD2DN. After the preparative cloning steps, the whole recombination cassette transferred to the suicide vector pPR28A to obtain the final construct pPRD2KO. The sucrose-resistant, gentamicin-sensitive and kanamycin-resistant colonies were selected for further analysis. We have verified the disruption of the gene and the recombination event by PCR and Southern hybridization technique as standardized in our laboratory [45].

Four types of mycobacterial strains [wild type, over expressed MsDps2, over expressed MsDps1, Knockout MsDps2 (KK09)] were grown in different time intervals (12 h, 24 h, 48 h, 72 h, and 144 h) in both 2% and 0.02% glucose. The bacterial growth was standardized to a uniform optical density of 0.5 for each strain and then the cell cultures were centrifuged at 6000 rpm for 20 min.

### Lysis procedure

After centrifugation, 200 µl of lysis buffer [consisting of 50 mM Tris-Cl (pH 7.4), 50 mM NaCl, 1 mM PMSF, 0.1% Triton x-100, 2 mM βME] were added to the cells, followed by sonication at 2 min pulse interval. Then the samples were centrifuged again at 8000 rpm for 20 min at 4°C. The supernatant was dialyzed against 40 mM HEPES and used for further analysis.

### RNaseA Treatment

RNaseA treatment at desired concentration (0.5 mg/ml, 1 mg/ml, 2 mg/ml and 5 mg/ml) were carried out on 2 µl of cell lysate with protein concentration of 150 µg/ml and a buffer [Buffer (I)] containing 60 mM HEPES and 10 mM NiCl_2_ and 10 mM MgCl_2_. After RNaseA treatment, presence of Dps2 in lysate was confirmed by western analysis. Subsequently this lysate was used for AFM analysis. To generate the antibody against MsDps2 we followed the same protocol as discussed in previous report [21].

### Effect of Mg^2+^ ions

In our laboratory, for this study, we used and standardized one buffer, which helps to get AFM images with a good resolution. We have named it Buffer (I), which contains mainly HEPES, NiCl_2_ and MgCl_2_. Here both NiCl_2_ and MgCl_2_ help to attach the sample to the mica surface.

Initially 10 mM MgCl_2_ was added to the Buffer (I) which also contained 60 mM HEPES and 10 mM NiCl_2_. Same amount of lysate of various strains of mc^2^155 were added in this buffer. However, in order to check for the effect of magnesium ion on nucleoid structure, 1 mM and 7.5 mM MgCl_2_ were separately added to this Buffer (I) instead of 10 mM MgCl_2_. The amount of HEPES and NiCl_2_ were the same in all cases.

### Protein Purification

Both the proteins MsDps1 and MsDps2 were purified by following the procedure for MsDps2 as described previously [20].

### Rifampicin treatment

mc^2^155 cells, transformed with plasmid (pAGAN *msdps2*) containing *msdps2* gene were over expressed. They were grown in MB7H9 medium supplemented with 0.02% glucose and harvested up to 72 h. Then the cells were treated with rifampicin (20 mg/ml), grown until 144 h. These cells were lysed as discussed above and subjected to AFM analysis.

### AFM analysis

AFM analysis has been done using the 5500 AFM imaging instrument from Agilent Technologies. For cell lysate analysis, we added 2 µl of lysate to Buffer (I), containing 60 mM HEPES, 10 mM NiCl_2_ and 10 mM MgCl_2_. Then we applied the reaction mixture on mica, after 5 min rinsed with 200 µl MilliQ water and dried in vacuum desiccator for 30 min. The instrument was operated in tapping mode at 22°C temperature using nanosensor tips (NCL type, spring constant 47–53 n/m, resonance frequency 267–298 KHz) and were taken in a 512/512 pixel format at a scan speed of 0.5–1 line/sec. Images were obtained and analyzed using the Picoimage software. Several measurements were taken and the mean dimensions with standard deviation are given.

### Genotypic confirmation of *msdps2* knockout by Southern hybridization

All the primers, used in this study have been listed in Table S1.

The schematic representations of knockout construct (Figure S7), PCR confirmation of the knockout strain is shown in Supplementary Figure S8 and S9. The whole process of construction of Dps2 knockout strain or KK09 has been described in Text S2. The genotypic confirmation of the knockout mutation was substantiated by Southern hybridization. The genomic DNA of the putative mutant as well as *M. smegmatis* mc^2^155 was digested with *Apa*I and subjected to Southern hybridization. The probe used was approximately 1.2 kb long DNA fragment conferring kanamycin resistance; *kan* ^r^ excised from pUC4K by digesting with *Eco*RI. The probe was labeled with [α^32^P] ATP using the random primer labeling kit (Bangalore Genei, India) following the manufacturer's protocol. Southern hybridization was carried out as mentioned [46]. After Southern hybridization, Hybond-XL membrane was exposed to phosphorimager screens and analyzed (PhosphorImager, Molecular Dynamics). Upon digestion with *Apa*I, the probe hybridized to an approximately 4.4 kb long fragment in the mutant indicating the insertion of kanamycin cassette to disrupt the *msdps2* gene, whereas in case of parental strain, hybridization did not take place due to the absence of gene disruption (Figure S10) [45].

## Supporting Information

Figure S1 **AFM images of mycobacterial nucleoid.** (A) Compaction of nucleoid structures and (B) intact nucleoid structures. Cells were taken from over expressed Dps2 strain grown up to 170 h and 220 h respectively, in 0.02% glucose. The diameters are 2.5 µm±0.4 µm and 2 µm±0.5 µm (structure and area of nucleoid are matched with previous reports).
(TIF)

Figure S2 **AFM images of the cellular extract of four strains of *M. smegmatis* cells, grown at 12 h, 24 h, 48 h, 72 h and 144 h. **All images were taken as described in Materials and methods. The blobs represent multimeric protein complex and the linear network at the background is due to DNA. (A) AFM images of the cellular extract of *M. smegmatis* wild-type cells grown in 2% glucose at 12 h, 24 h, 48 h, 72 h and 144 h. (B) AFM images of the cellular extract of *M. smegmatis* wild-type cells grown in 0.02% glucose at 12 h, 24 h, 48 h, 72 h and 144 h. (C) AFM images of the cellular extract of over expressed MsDps1 cells grown in 0.02% glucose at 12 h, 24 h, 48 h, 72 h and 144 h. (D) AFM images of the cellular extract of over expressed MsDps2 cells grown in 0.02% glucose at 12 h, 24 h, 48 h, 72 h and 144 h. (E) AFM images of the cellular extract of knockout MsDps2 (KK09) cells grown in 0.02% glucose at 12 h, 24 h, 48 h, 72 h and 144 h.
(TIF)

Figure S3 **AFM images of coral reef structure before and after Immunoprecipitation of cellular extract of mc^2^155 cells grown in 0.02% glucose at 144 h.** (A–B) shows coral reef structure which are formed by over expressed MsDps2 cell extract before and after Immunoprecipitation. (C–D) shows nucleoid without coral reef structure, which was formed by over expressed MsDps1 cell lysate before and after Immunoprecipitation.
(TIF)

Figure S4 **AFM images of the cellular extracts made from mc^2^155 cells over expressing Dps2 grown till 144 h, after treatment with rifampicin in growing cells at 72 h (absence of coral reef structure).**
(TIF)

Figure S5 **AFM images of the cellular extracts of over expressed MsDps1. **(A) without RNaseA treatment. (B) Treated with 5 mg/ml RNaseA in 0.02% glucose at 144 h.
(TIF)

Figure S6 **Nucleoid structures from over expressed Dps1, KK09 and over expressed Dps2 strains, grown up to 144 h at 0.02% (in the presence of MgCl_2_ at different concentration).** A (1–3) Molecular complex appeared with the addition of 1 mM [Figure A1] and 7.5 mM MgCl_2_ [Figure A2]. However, this complex more or less disappears in the presence of 10 mM MgCl_2_ [Figure A3]. B (1–3) Nucleoid structures from knockout Dps2 strain, grown up to 144 h in 0.02% glucose. DNA- protein complex shows more condensed structure in the presence of 1 mM (Figure B1) and 7.5 mM [Figure B2] MgCl_2_ than that of in 10 mM MgCl_2_ [Figure B3]. C (1–3) AFM images of nucleoid structures of over expressed Dps2 strain grown up to 144 h at 0.02% glucose. There is no effect of Mg^2+^ ions on coral reef structure at various concentrations like 1 mM [Figure C1], 7.5 mM [Figure C2] and 10 mM [Figure C3].
(TIF)

Figure S7 **A schematic representation of all the steps involved in the construction of the recombination cassette for the disruption of *msdps*2 in *M. smegmatis* using *kan*^r^ (*aph*) gene.** The sizes of the vectors are approximate estimations of their size. Construction procedure of KK09 has been described in Text S2.
(TIF)

Figure S8 **1% agarose gel showing the products of colony PCR with primers MsDps2fwd and MsDps2rev.** Lane M: 1 kb Gene ruler ladder, Lane 1: colony PCR with clone showing amplification of both 509 bp and 1.4 kb bands, Lane 2: colony PCR with wild type shows a 509 bp band, Lane 3: PCR with genomic DNA from wild type yields a 509 bp band (Text S2).
(TIF)

Figure S9 1**% agarose gel showing the products of colony PCR with primers Dps2KJForKO and KanF with clone and wild type *M. smegmatis.*** Lane M: 1 kb Gene ruler ladder, Lane 1: Control colony PCR with wild type strain, Lane 2: colony PCR with clone showing amplification of 1.63 kb band (Text S2).
(TIF)

Figure S10 **Genotypic confirmation of *msdps*2 knockout by southern hybridization.** Lane M: 1 kb Gene ruler ladder (Fermentas), Lanes 1 and 2: 4.4 kb band after southern hybridization confirming the insertion of *kan* ^r^ gene in the mutant after partial disruption of *msdps2*, Lane 3: Absence of band indicating no mutation in the wild type.
(TIF)

Table S1 Primers used in this study.
(DOC)

Text S1 Immunoprecipitation.
(DOC)

Text S2 Construction of a disruption mutation of *msdps*2 in *M. smegmatis.*
(DOC)
