# Supplementary figures and images for: Unveiling the Role of Dps in the Organization of Mycobacterial Nucleoid
Source: PLoS One. 2011 Jan 24;6(1):e16019. doi: 10.1371/journal.pone.0016019 (PMC3026007; doi:10.1371/journal.pone.0016019)

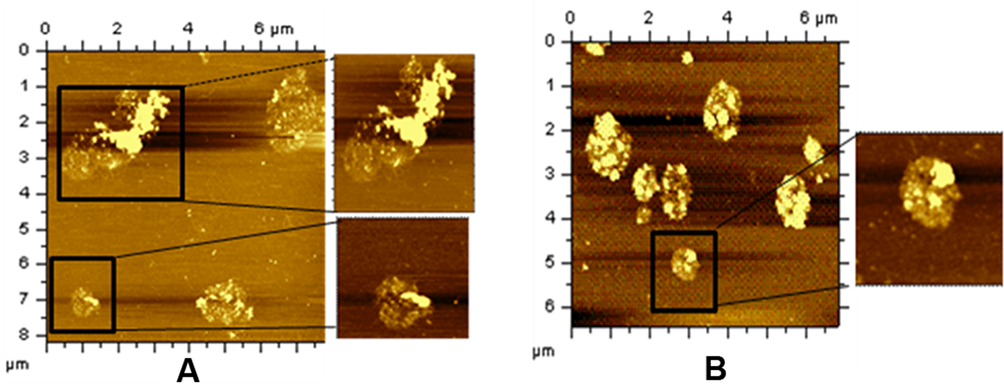

Supplement: Figure S1 — AFM images of mycobacterial nucleoid. (A) Compaction of nucleoid structures and (B) intact nucleoid structures. Cells were taken from over expressed Dps2 strain grown up to 170 h and 220 h respectively, in 0.02% glucose. The diameters are 2.5 µm±0.4 µm and 2 µm±0.5 µm (structure and area of nucleoid are matched with previous reports). (TIF) [file pone.0016019.s001.tif]

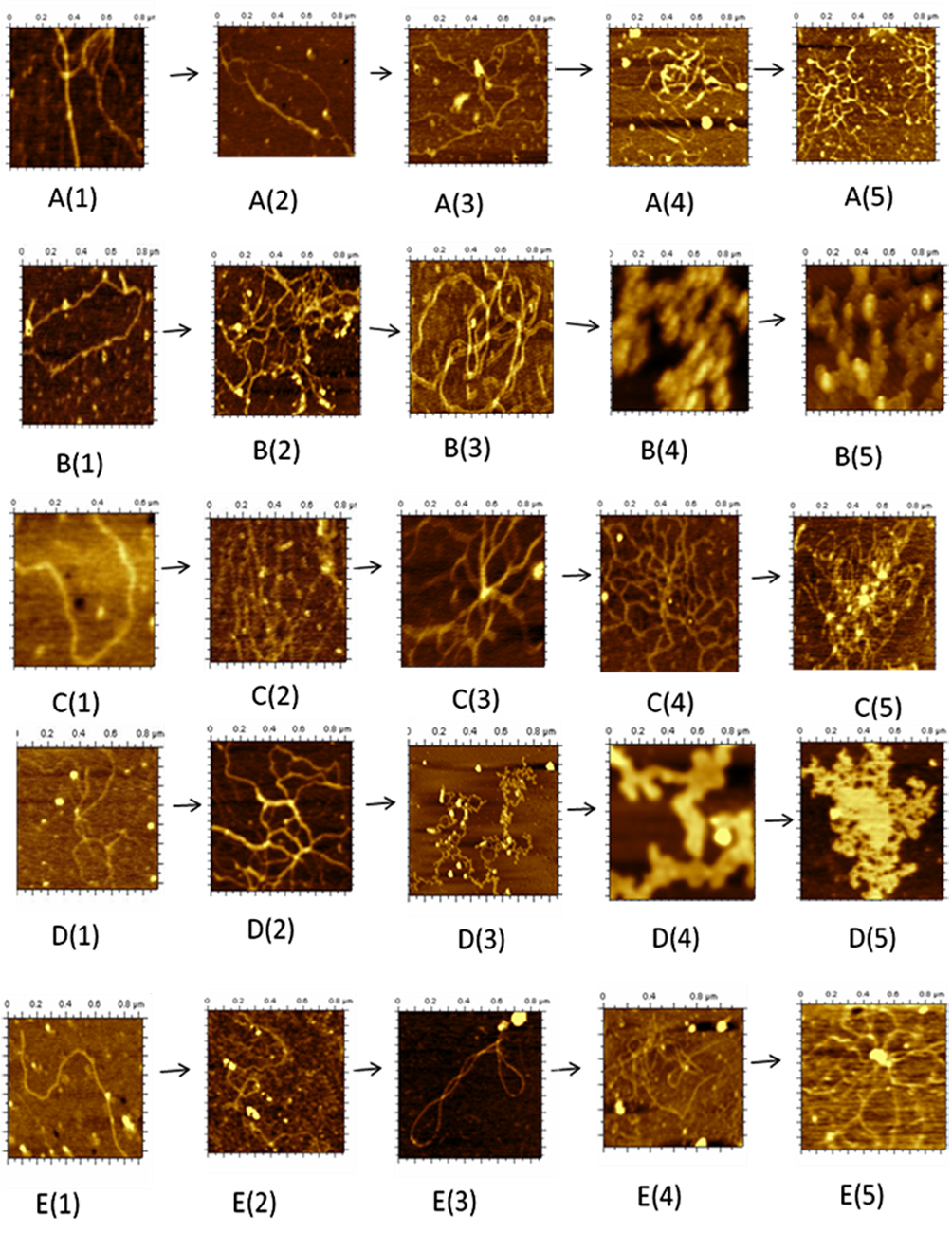

Supplement: Figure S2 — AFM images of the cellular extract of four strains of M. smegmatis cells, grown at 12 h, 24 h, 48 h, 72 h and 144 h. All images were taken as described in Materials and methods. The blobs represent multimeric protein complex and the linear network at the background is due to DNA. (A) AFM images of the cellular extract of M. smegmatis wild-type cells grown in 2% glucose at 12 h, 24 h, 48 h, 72 h and 144 h. (B) AFM images of the cellular extract of M. smegmatis wild-type cells grown in 0.02% glucose at 12 h, 24 h, 48 h, 72 h and 144 h. (C) AFM images of the cellular extract of over expressed MsDps1 cells grown in 0.02% glucose at 12 h, 24 h, 48 h, 72 h and 144 h. (D) AFM images of the cellular extract of over expressed MsDps2 cells grown in 0.02% glucose at 12 h, 24 h, 48 h, 72 h and 144 h. (E) AFM images of the cellular extract of knockout MsDps2 (KK09) cells grown in 0.02% glucose at 12 h, 24 h, 48 h, 72 h and 144 h. (TIF) [file pone.0016019.s002.tif]

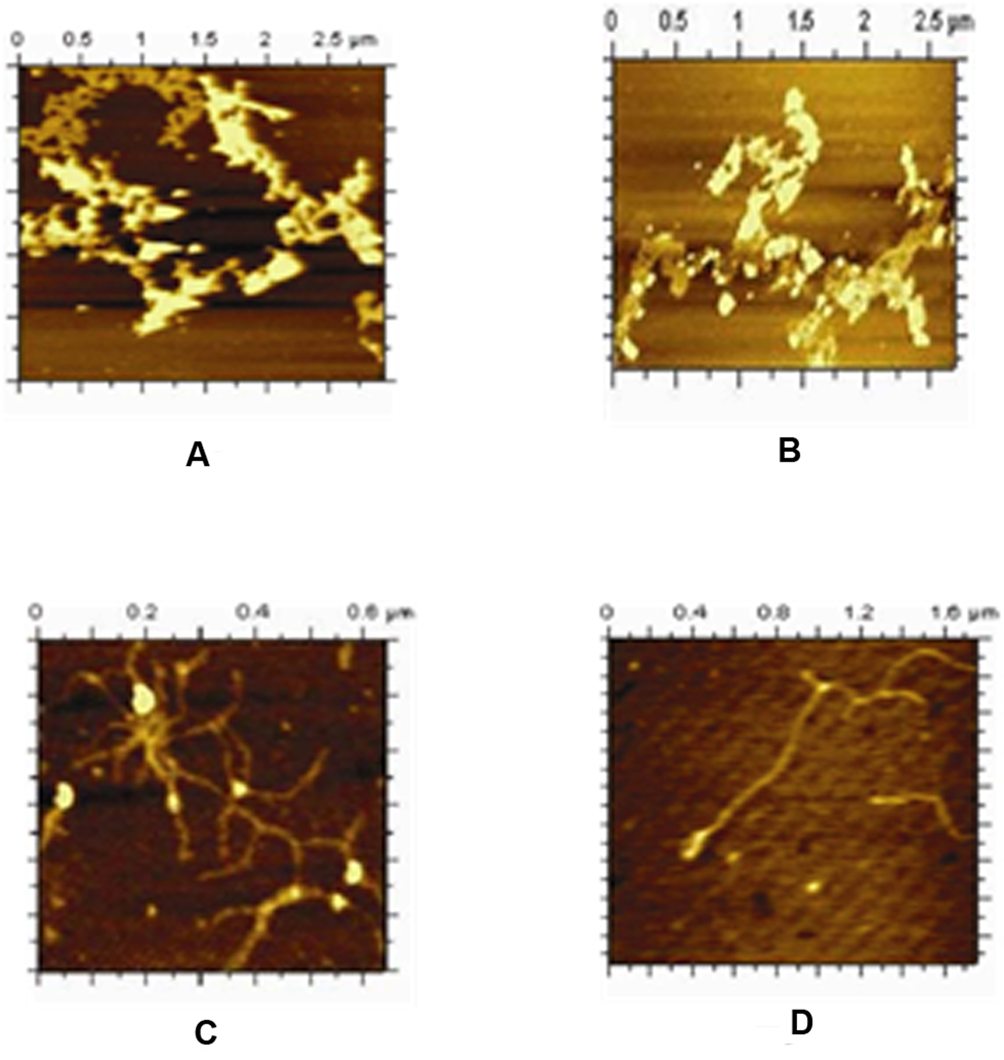

Supplement: Figure S3 — AFM images of coral reef structure before and after Immunoprecipitation of cellular extract of mc2155 cells grown in 0.02% glucose at 144 h. (A–B) shows coral reef structure which are formed by over expressed MsDps2 cell extract before and after Immunoprecipitation. (C–D) shows nucleoid without coral reef structure, which was formed by over expressed MsDps1 cell lysate before and after Immunoprecipitation. (TIF) [file pone.0016019.s003.tif]

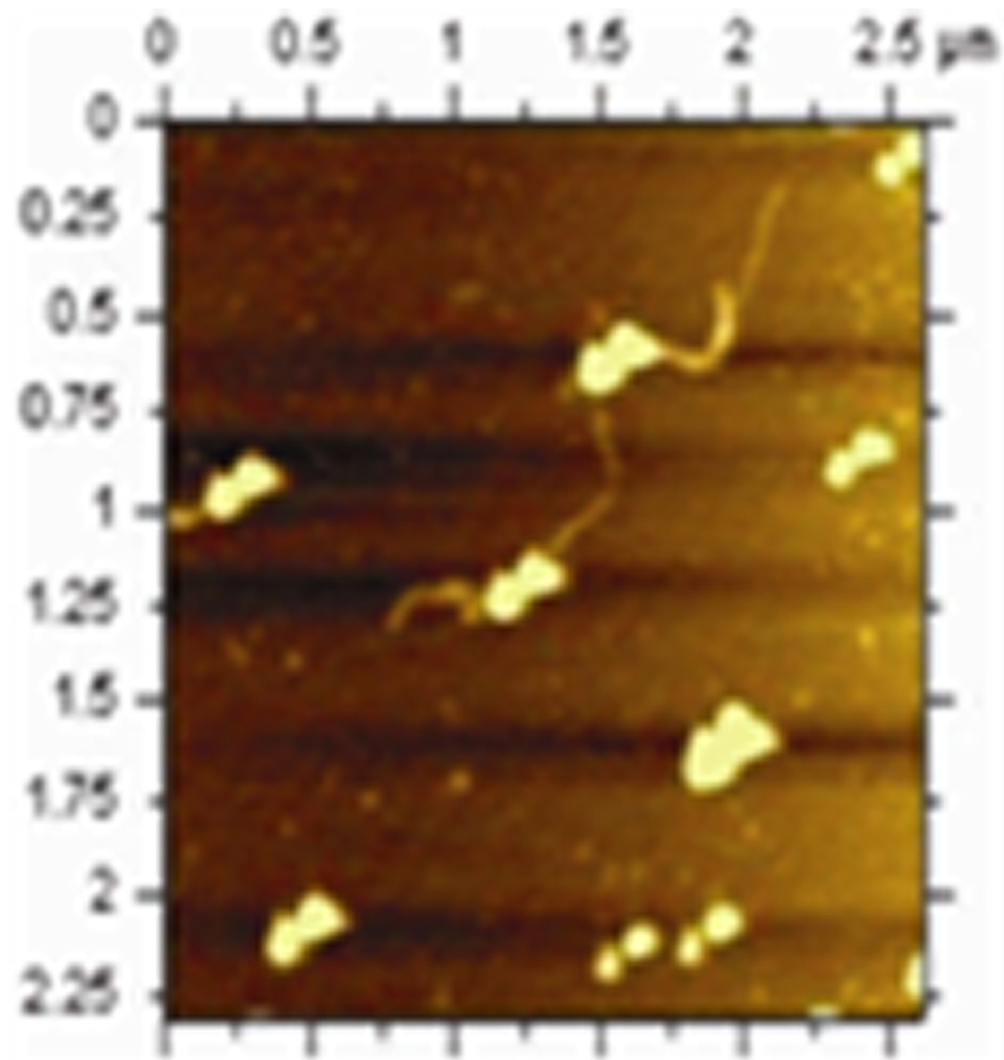

Supplement: Figure S4 — AFM images of the cellular extracts made from mc2155 cells over expressing Dps2 grown till 144 h, after treatment with rifampicin in growing cells at 72 h (absence of coral reef structure). (TIF) [file pone.0016019.s004.tif]

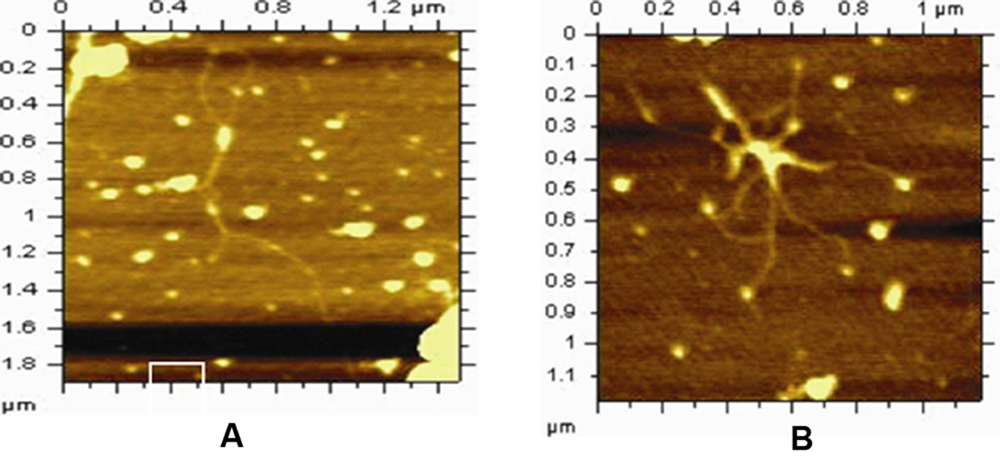

Supplement: Figure S5 — AFM images of the cellular extracts of over expressed MsDps1. (A) without RNaseA treatment. (B) Treated with 5 mg/ml RNaseA in 0.02% glucose at 144 h. (TIF) [file pone.0016019.s005.tif]

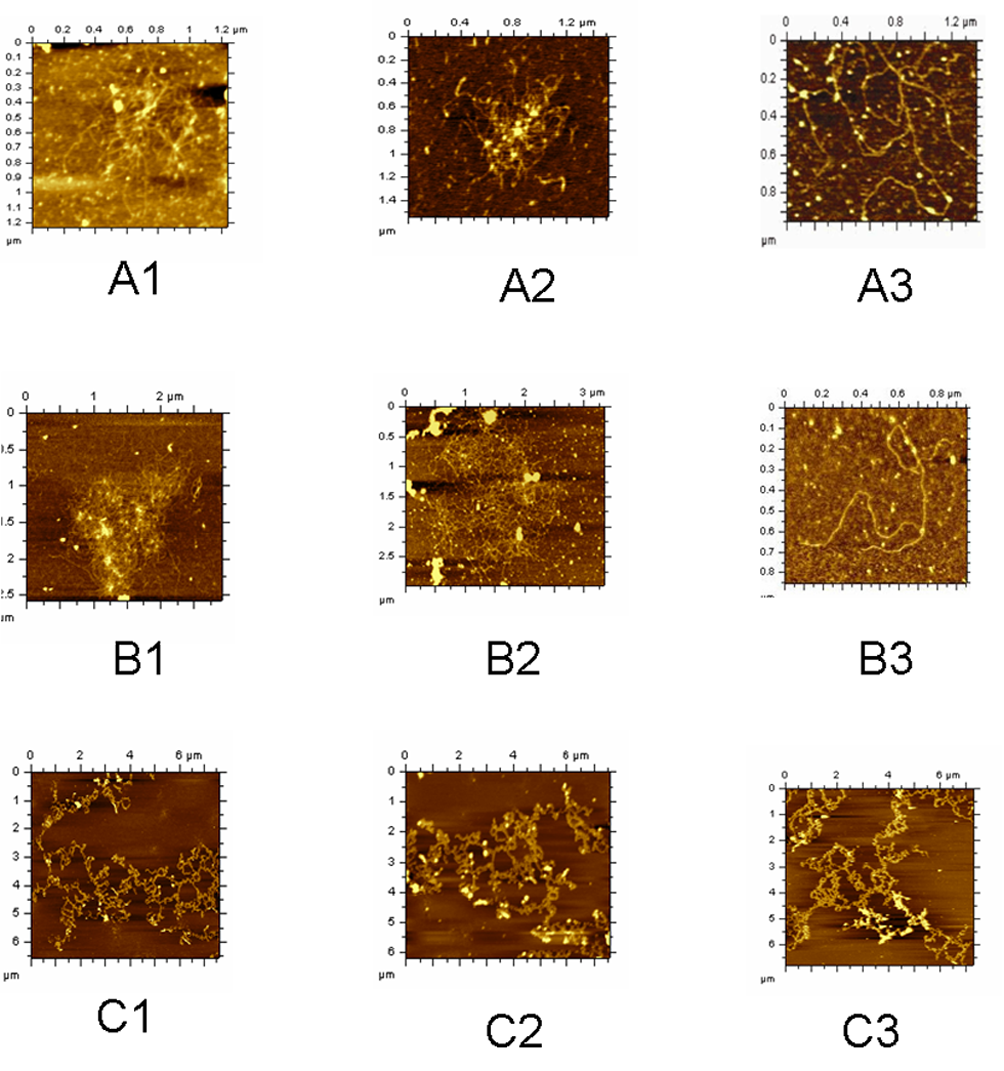

Supplement: Figure S6 — Nucleoid structures from over expressed Dps1, KK09 and over expressed Dps2 strains, grown up to 144 h at 0.02% (in the presence of MgCl2 at different concentration). A (1–3) Molecular complex appeared with the addition of 1 mM [Figure A1] and 7.5 mM MgCl2 [Figure A2]. However, this complex more or less disappears in the presence of 10 mM MgCl2 [Figure A3]. B (1–3) Nucleoid structures from knockout Dps2 strain, grown up to 144 h in 0.02% glucose. DNA- protein complex shows more condensed structure in the presence of 1 mM (Figure B1) and 7.5 mM [Figure B2] MgCl2 than that of in 10 mM MgCl2 [Figure B3]. C (1–3) AFM images of nucleoid structures of over expressed Dps2 strain grown up to 144 h at 0.02% glucose. There is no effect of Mg2+ ions on coral reef structure at various concentrations like 1 mM [Figure C1], 7.5 mM [Figure C2] and 10 mM [Figure C3]. (TIF) [file pone.0016019.s006.tif]

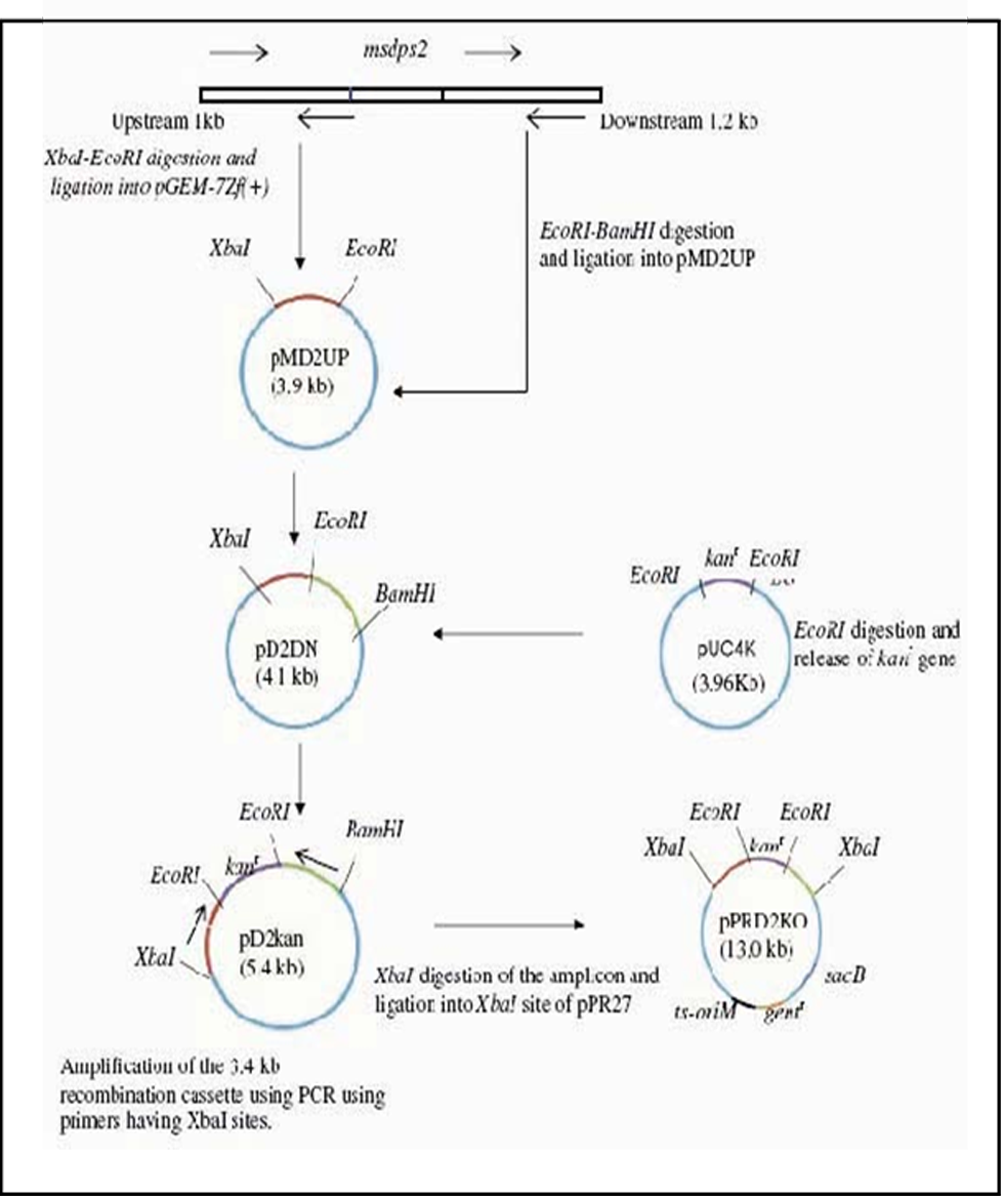

Supplement: Figure S7 — A schematic representation of all the steps involved in the construction of the recombination cassette for the disruption of msdps2 in M. smegmatis using kanr (aph) gene. The sizes of the vectors are approximate estimations of their size. Construction procedure of KK09 has been described in Text S2. (TIF) [file pone.0016019.s007.tif]

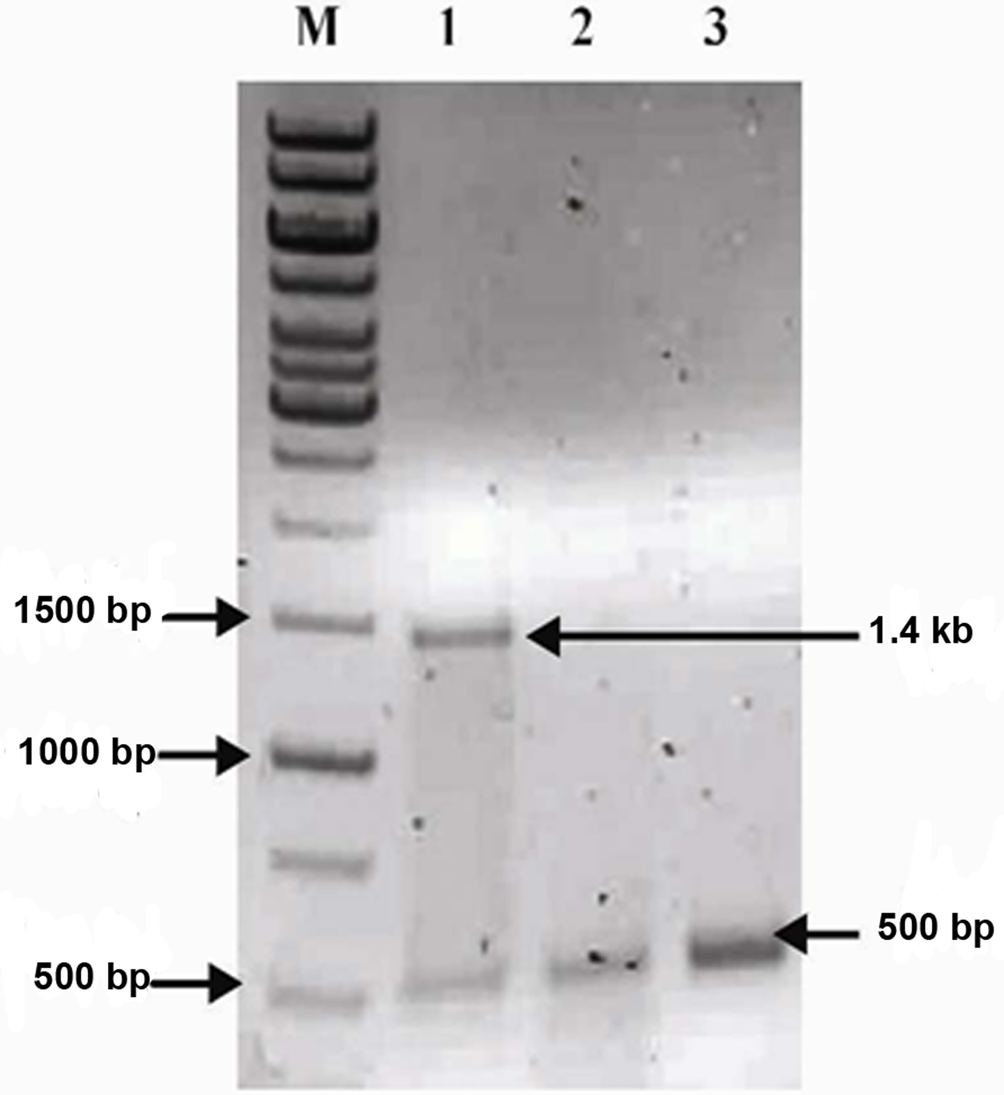

Supplement: Figure S8 — 1% agarose gel showing the products of colony PCR with primers MsDps2fwd and MsDps2rev. Lane M: 1 kb Gene ruler ladder, Lane 1: colony PCR with clone showing amplification of both 509 bp and 1.4 kb bands, Lane 2: colony PCR with wild type shows a 509 bp band, Lane 3: PCR with genomic DNA from wild type yields a 509 bp band (Text S2). (TIF) [file pone.0016019.s008.tif]

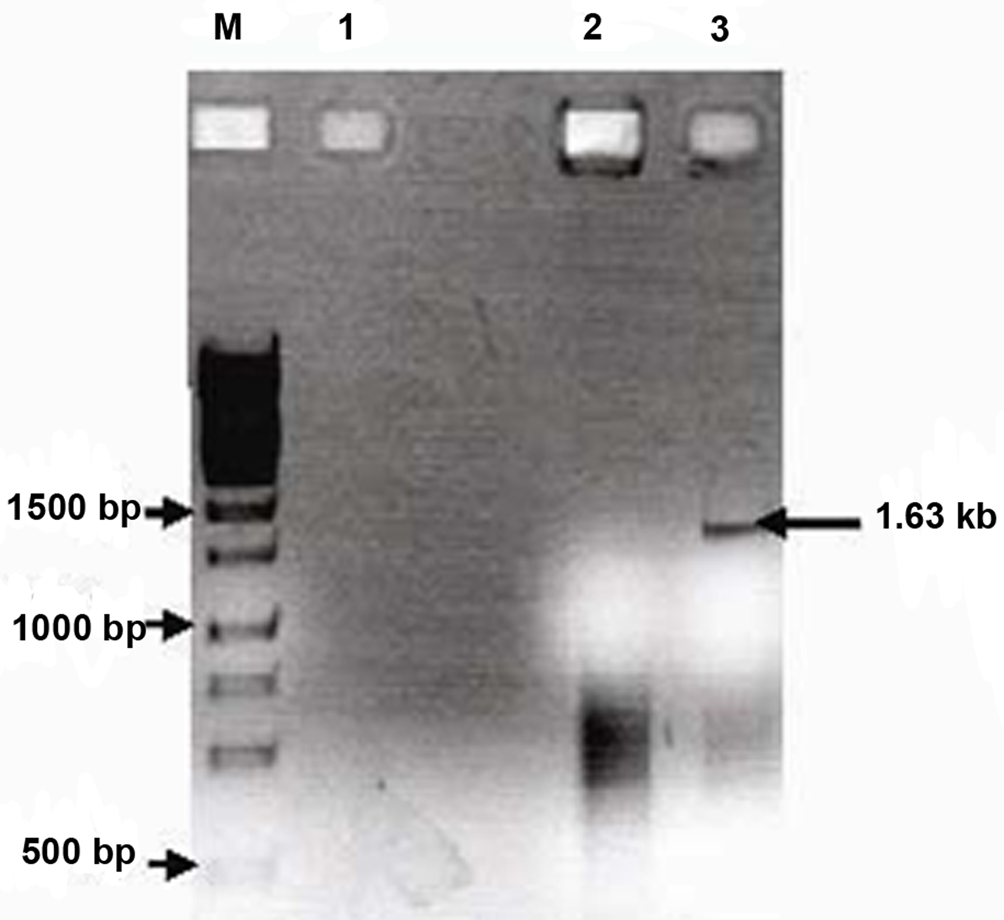

Supplement: Figure S9 — 1% agarose gel showing the products of colony PCR with primers Dps2KJForKO and KanF with clone and wild type M. smegmatis. Lane M: 1 kb Gene ruler ladder, Lane 1: Control colony PCR with wild type strain, Lane 2: colony PCR with clone showing amplification of 1.63 kb band (Text S2). (TIF) [file pone.0016019.s009.tif]

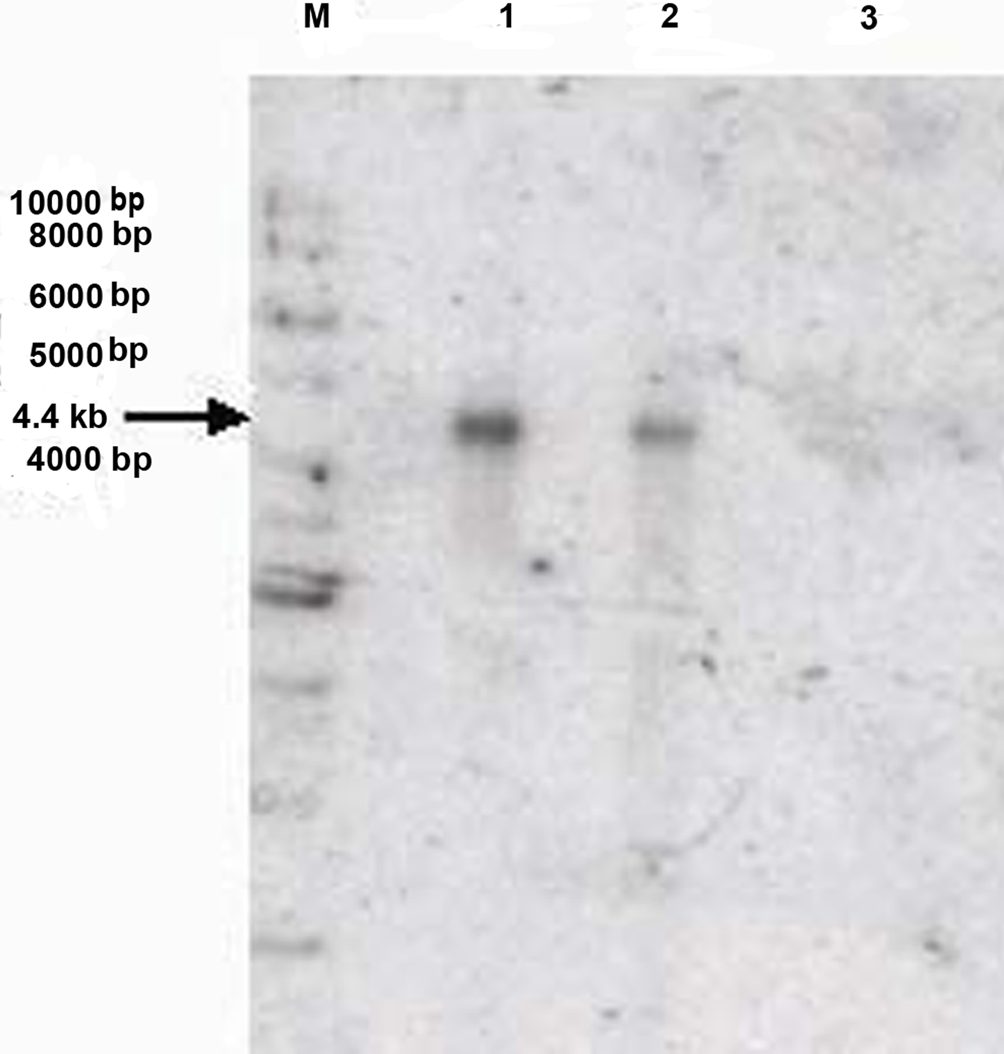

Supplement: Figure S10 — Genotypic confirmation of msdps2 knockout by southern hybridization. Lane M: 1 kb Gene ruler ladder (Fermentas), Lanes 1 and 2: 4.4 kb band after southern hybridization confirming the insertion of kan r gene in the mutant after partial disruption of msdps2, Lane 3: Absence of band indicating no mutation in the wild type. (TIF) [file pone.0016019.s010.tif]
