# Supplementary material for: Unveiling the Role of Dps in the Organization of Mycobacterial Nucleoid
Source: PLoS One. 2011 Jan 24;6(1):e16019. doi: 10.1371/journal.pone.0016019 (PMC3026007; doi:10.1371/journal.pone.0016019)
Supplement: Table S1 — Primers used in this study. (DOC) [file pone.0016019.s011.doc]

**Table S1**

**Primers used in this study**

| **Primers** | **Sequence** | **Restriction sites** |
| --- | --- | --- |
| Dps2KJForKO | 5' ACCGTGGTCTAGACGCTGGG 3' | *Xba*I |
| Dps2KJRevKO | 5' TGCCGCCGAATTCCGGTGTGGCATGG 3' | *Eco*RI |
| NWD2DNFor | 5’GTGGACGCCGAGAATTCCAGCACGGCCGATC3’ | *Eco*RI |
| NWD2DNRev | 5’ CCACGCTCACTTGGATCCTCCATTGTGCC 3’ | *Bam*HI |
| pPRD2KOFor | 5' TGCCGTCCTCTAGATCGCCGGGCTGGCGG 3' | *Xba*I |
| pPRD2KORev | 5' GTCCGCGCTCGGTCTAGATGCCCGAACTGG 3' | *Xba*I |
| MsDps2For | 5’ CAGAAAAGCTAGCGTTGGAC 3’ | *Nhe*I |
| MsDps2Rev | 5’GGCGGTTAGAAGCTTAGACC 3’ | *Hind*III |
| KanF | 5’ GCCTGAGCGAGACGAAATACGCGATCG 3’ | **-** |
