# Supplementary material for: Unveiling the Role of Dps in the Organization of Mycobacterial Nucleoid
Source: PLoS One. 2011 Jan 24;6(1):e16019. doi: 10.1371/journal.pone.0016019 (PMC3026007; doi:10.1371/journal.pone.0016019)
Supplement: Text S1 — Immunoprecipitation. (DOC) [file pone.0016019.s012.doc]

**Text S1**

**Immunoprecipitation.** In order to confirm that MsDps2 is the only protein which participates in coral reef formation we have carried out immunoprecipitation in the presence of both MsDps1 and MsDps2. In immunoprecipitation technique NHS-column

was used as matrix. At first, the column was activated by 1 mM HCl. Then the purified antibody against MsDps2 was added to the column. After 72 h and 144 h over expressed MsDps2 and MsDps1 cell lysate were passed through the different NHS column separately. For elution, glycine (pH 2.9) was used. The eluted sample was collected and used for AFM analysis [Figure S3 (A-D)].
