# Supplementary material for: Unveiling the Role of Dps in the Organization of Mycobacterial Nucleoid
Source: PLoS One. 2011 Jan 24;6(1):e16019. doi: 10.1371/journal.pone.0016019 (PMC3026007; doi:10.1371/journal.pone.0016019)
Supplement: Text S2 — Construction of a disruption mutation of msdps2 in M. smegmatis. (DOC) [file pone.0016019.s013.doc]

**Text S2**

**Construction of a disruption mutation of *msdps*2 in *M. smegmatis***

**Construction of a suicide delivery vector for the replacement of *msdps*2 with *kan*^r^ using pPR27 vector.** The steps involved in the construction of the suicide delivery vector bearing the recombination cassette to replace the chromosomal copy of *msdps*2 have been shown in a schematic diagram (Figure S7). The principle of the construction was based on allelic exchange mutagenesis in *M. smegmatis*. The vector system used - pPR27 is a mycobacterial shuttle vector, with an *E. coli* origin of replication, *oriE* and a thermo-sensitive Mycobacterial origin of replication, *ts-oriM* (1). So the vector survives in mycobacteria at 30°C or below, but is incapable of replicating at temperatures of 39°C or above. The vector also carries gentamicin resistance gene, *gent*^r^ and a *sacB* gene. The *sacB* gene when expressed renders the cells harboring the vector lethal in the presence of sucrose. Thus, *sacB* and ts-oriM provide efficient counter selective measures for identification of the mutants.

**Cloning of the upstream 1kb region of *msdps2* into pGEM-7Zf (+) vector.** A DNA fragment ranging from the 966th base upstream of the *msdps2* gene to 64th base into the gene was amplified by Polymerase Chain Reaction, using the primers Dps2KJForKO and Dps2KJRevKO (2). The PCR reaction contained Primers 0.2 μM, dNTPs 0.2 mM, Template DNA 50-100 ng, DMSO 5%. The parameters for thermal cycling reactions in the PCR are; Initial denaturation, 95°C for 5 min; denaturation, 95°C for 1 min; annealing, 56.2°C for 1 min; extension, 72°C for 4 min for 29 cycles. The amplified PCR product was digested with *Xba*I and *Eco*RI (New England Biolabs) and cloned into the vector pGEM-7Zf (+) to yield pMD2UP. Competent *E. coli* DH5α cells were transformed with the ligated product and the clones were screened on LB-agar plates containing ampicillin (100 μg/ml). The clones were confirmed using restriction digestions to release the insert.

**Cloning of the 1.2 kb region downstream of *msdps2* into pMD2UP.** A DNA segment ranging from the 384th base of the *msdps2* gene to 1126th base downstream of the gene was amplified using the primers NWD2DNFor and NWD2DNRev. The PCR conditions were same as described earlier. The amplified product was digested with *Eco*RI and *Bam*HI and ligated into *Eco*RI*-Bam*HI digested pMD2UP to generate pD2DN. The clones were verified similarly as in the previous case.

**Cloning of *kan*^r^ gene into pD2DN.** The vector pUC4K (Pharmacia Biotech) was digested with *Eco*RI to release 1282 bp long *kan*^r^ gene insert*.* The insert was purified and ligated into the unique *Eco*RI site of pD2DN. The clones were confirmed by checking the gain of resistance to kanamycin (25 μg/ml) by the ampicillin resistant parent vector; along with restriction digestions.

**Cloning of the recombination cassette into pPR27.** pPR27 provides very little option of restriction sites to choose from for cloning into it. Due to the presence of most of the unique restriction sites of pPR27 within the recombination cassette, the entire construct was amplified by PCR using the primers pPRD2KOFor and pPRD2KORev, each carrying *Xba*I site and then cloned into pPR27. Amplification was carried out from 901st base upstream of *msdps2* start site to 1000^th^ base downstream of the stop codon. Mycobacterial genome being rich in GC-content often assumes a strong secondary structure in certain parts that provide a barrier to Polymerase chain reaction. Phusion DNA polymerase (Finnzymes) was used in this amplification with modified reaction conditions. The parameters for thermal cycling reactions in the PCR are; Initial denaturation, 95°C for 5 min; denaturation, 95°C for 1 min; annealing, 65°C for 1 min; extension, 72°C for 4 min for 29 cycles. The ~3.3 kb PCR amplified product was digested with *Xba*I (New England Biolabs) and inserted into the unique *Xba*I site of pPR27 to generate the suicide delivery vector pPRD2KO. The construct was then confirmed by sequencing using insert specific primers (MWG sequencing, Bangalore).

**Transformation of *M. smegmatis* with pPRD2KO.** Transformation of *M. smegmatis* was done by electroporation (3) 1-5 μg of DNA was added to electrocompetent *M. smegmatis* cells and incubated on ice for 5 min. It was then introduced into Bio-rad cuvettes and subjected to electroporation with Bio-rad electroporator at 1.5 kV/mm. The cells were then mixed with 5 ml liquid MB7H9 containing 2% glucose and 0.05% Tween 80, for recovery and grown till 6 h at 30°C. 1 ml of the culture was pelleted after 6 h and plated onto MB7H9 agar plates with gentamicin (10 μg/ml) and 2% glucose. The plates were incubated at 30°C for 5 days.

**Screening for clones with targeted disruption of *msdps*2 with *kan*^r^ in *M. smegmatis*.** The colonies obtained after transformation were plated on gentamicin (10 μg/ml) and 2% glucose containing MB7H9 agar plate and grown at 30°C to obtain patches. Colonies from one of the patches were inoculated into liquid MB7H9 medium containing kanamycin (25 μg/ml), 2% glucose and 0.05% Tween-80 and grown in a shaker incubator at 30°C for 36 h. This step was essential to increase the plasmid copy number in the cells and also to allow for allelic exchange to take place. In order to screen for double recombinants, the culture was plated onto MB7H9 agar plates containing 2% glucose, 0.05% Tween 80, 10% sucrose and kanamycin (25 μg/ml) at different dilutions and incubated at 39°C. The colonies obtained on these plates were then screened for gentamicin sensitivity by plating on MB7H9 agar plates with glucose, sucrose, and gentamicin (10 μg/ml). Effectively, colonies exhibiting kanamycin resistance, sucrose resistance and gentamicin sensitivity should be the double recombinants and hence the *msdps2* disruption mutants. The single crossovers would be expected to exhibit gentamicin resistance and sucrose sensitivity. However, 10% sucrose was seen to be ineffective in killing the plasmid bearing colonies and did not provide any selection. So the sucrose concentration was required to be standardized. Similarly, the gentamicin concentration was also standardized to verify that the gentamicin sensitivity was not an artifact due to overdose of gentamicin in single crossovers bearing a single copy of the *gent*^r^ gene but due to loss of *gent*^r^ in the double recombinants .

**Genotypic confirmation**

**PCR analysis for identification of *msdps*2 disruption mutants**

**MsDps2For and MsDps2Rev.** Initial screening was done with *msdps2* gene specific primers, MsDps2For and MsDps2Rev. Since the target was to generate a gene disruption mutation rather than a complete deletion, hence some region of the *msdps2* gene from the beginning and the end were amplified as part of the upstream and downstream regions respectively. Thus, these gene specific primers would amplify the region between them. In the case of a successful targeted disruption of *msdps2*, the interspersed gene would be 1282 bp long *kan*^r^ with 60 bp from the upstream region and 99 bp from the downstream region of *msdps2*, yielding a product of 1450 bp. In case of non recombinants, the *msdps2* gene would be amplified and the amplicon size would be 509 bp. In case of single crossover events or mixed populations of cells, both the amplicons corresponding to 1450 bp and 509 bp would be obtained. The PCR was carried out with Taq DNA Polymerase (Bangalore Genei, India). The PCR reaction mixture was same as mentioned earlier except that no DMSO was added to the mixture and the buffer used was Taq Buffer A (containing MgCl_2_). The thermal cycling parameters were kept same as mentioned earlier except that the annealing temperature was kept at 53.9°C for 1 min and the time for extension was decreased to 2 min.

**Dps2KJForKO and KanF.** The above amplification would also take place if the recombination occurs at any non-specific site. So, this set of primers was used, such that Dps2KJForKO is 966th bases upstream and is outside the construct, whereas, KanF is a reverse primer specific to the kanamycin gene. This amplification would only take place if the recombination takes place at the desired site. The expected amplicon size is 1627 bp in the disruption mutant. The PCR was carried out with DyNAzyme DNA Polymerase using the same thermal cycling parameters as mentioned earlier except that the annealing temperature was set at 57°C for 1 min.

**MsDps2For and NWD2DNRev.** This combination of primers would result in the amplification of a 2580 bp long amplicon in case of the double recombinant mutant whereas, in the wild type or single crossovers, it will produce a product of 1.7 kb. Since this reaction will work for both double recombinants and wild type, so it is easy to standardize and also troubleshoot. Moreover, the result of this reaction will also be a clear indication of the site of recombination as MsDps2For is specific to the construct whereas, NWD2DNRev binds to a genomic region outside the construct. The reaction was carried out with DyNAzyme DNA Polymerase using its specific buffer in presence of 5% DMSO. The PCR conditions were same as mentioned earlier except that the annealing temperature was kept at 57°C for 1 min.

**Results**

**Screening of *msdps2* disruption mutants using PCR.** Using the PCR reactions mentioned above, the putative clones were screened. The clones exhibiting gentamicin sensitivity were screened with colony PCR using the primers MsDps2For and MsDps2Rev. The resultant PCR products when ran on 1% agarose gel, showed the presence of the 509 bp band corresponding to the *msdps2* gene. However, in some of the clones an additional 1.4 kb band corresponding to the expected result in case of a successful disruption event was also obtained (Figure S8). Using the primers Dps2KJForKO and KanF which are *aph* gene specific will amplify *msdps2* and will not amplify in the wild type strain (Figure S9). This confirms it as a mutant strain with *msdps2* disrupted with *aph* or *kan*^r^.

**References**

1. Pelicic V, Jackson M, Reyrat JM, Jacobs WR Jr, Gicquel B, et al. (1997) Efficient allelic exchange and transposon mutagenesis in Mycobacterium tuberculosis. Proc Natl Acad Sci USA 94**:** 10955–10960.

2. Mullis K, Faloona F, Scharf S, Saiki R, Horn G, et al. (1986) Specific enzymatic amplification of DNA *in vitro*: the polymerase chain reaction. Cold Spring Harb Symp Quant Biol 51: 263–273.

3. Snapper SB, Melton RE, Mustafa S, Kieser T, Jacobs WR Jr (1990) Isolation and characterization of efficient plasmid transformation mutants of *Mycobacterium smegmatis*. Mol Microbiol 4: 1911**–**1919.
